# Turnaround Time for Early Infant HIV Diagnosis in Rural Zambia: A Chart Review

**DOI:** 10.1371/journal.pone.0087028

**Published:** 2014-01-24

**Authors:** Catherine G. Sutcliffe, Janneke H. van Dijk, Francis Hamangaba, Felix Mayani, William J. Moss

**Affiliations:** 1 Department of Epidemiology, Bloomberg School of Public Health, Johns Hopkins University, Baltimore, Maryland, United States of America; 2 Macha Research Trust, Macha Hospital, Choma, Zambia; 3 Department of Immunology and Infectious Diseases, Erasmus University Rotterdam, Rotterdam, the Netherlands; University of Cape Town, South Africa

## Abstract

**Background:**

Early infant HIV diagnosis is challenging in sub-Saharan Africa, particularly in rural areas where laboratory capacity is limited. Specimens must be transported to central laboratories for testing, leading to delays in diagnosis and initiation of antiretroviral therapy. This study was undertaken in rural Zambia to measure the turnaround time for confirmation of HIV infection and identify delays in diagnosis.

**Methods:**

Chart reviews were conducted from 2010–2012 for children undergoing early infant HIV diagnosis at Macha Hospital in Zambia. Relevant dates, receipt of drugs by mother and child for the prevention of mother-to-child transmission (PMTCT), and test results were abstracted.

**Results:**

403 infants provided 476 samples for early infant diagnosis. The median age at the “6-week” and “6-month” assessments was 8.1 weeks and 7.0 months, respectively. The majority of mothers (80%) and infants (67%) received PMTCT. The median time between sample collection and arrival at the central laboratory in Lusaka was 17 days (IQR: 10, 28); arrival at the central laboratory to testing was 6 days (IQR: 5, 11); testing to return of results to the clinic was 29 days (IQR: 17, 36); arrival of results at the clinic to return of results to the caregiver was 45 days (IQR: 24, 79). The total median time from sample collection to return of results to the caregiver was 92 days (IQR: 84, 145). The proportion of HIV PCR positive samples was 12%. The total median turnaround time was shorter for HIV PCR positive as compared to negative or invalid samples (85 vs. 92 days; p = 0.08).

**Conclusions:**

Delays in processing and communicating test results were identified, particularly in returning results from the central laboratory to the clinic and from the clinic to the caregiver. A more efficient process is needed so that caregivers can be provided test results more rapidly, potentially resulting in earlier treatment initiation and better outcomes for HIV-infected infants.

## Introduction

Over 90% of the world’s 3.3 million HIV-infected children reside in sub-Saharan Africa where resources for providing care and treatment are most limited [1]. Over the past decade, the capacity for providing antiretroviral treatment (ART) to HIV-infected individuals increased dramatically, with an estimated 387,500 children in sub-Saharan Africa receiving treatment at the end of 2010 [2]. While much progress has been made, this represents only 21% of children living with HIV in need of antiretroviral therapy, considerably lower than the estimated coverage of 55% among adults living with HIV [2].

One of the main factors for low coverage of ART among children is the large number of infants who remain undiagnosed but would be eligible for treatment under current treatment guidelines [3]. Diagnosis of HIV infection in early infancy is challenging in sub-Saharan Africa, as assays based on HIV DNA or RNA detection must be used rather than the serologic assays used in older children and adults [4]. These assays require a higher level of technology, training and cost than is available in many clinics and district laboratories. A high level of quality assurance also must be maintained as contamination with even a small amount of HIV nucleic acid can result in false positive results. Consequently, testing facilities are primarily available in major urban areas in centralized laboratories [5]. As a result of these challenges, only 15% of infants born to HIV-infected women in 2009 were estimated to have received early infant testing [6].

For HIV clinics in rural areas, where many HIV-exposed and infected infants reside, a centralized testing system poses significant challenges. Specimens must be transported to central laboratories and test results transported back to the clinic. Clinic appointments to provide results to caregivers need to be scheduled to account for the variability in the timing of this process, leading to delays in diagnosis and treatment. The World Health Organization (WHO) recommends that results be returned to caregivers within four weeks of specimen collection [3]. Data are emerging, however, that this process takes longer in rural areas of sub-Saharan Africa [7]–[10]. In a program in northern Tanzania with a regional laboratory serving four health facilities, the median turnaround time from blood draw to return of results to the caregiver was 5 weeks for HIV-infected infants and 10 weeks for uninfected infants [7].

To expedite early infant diagnosis and facilitate early linkage to care and treatment for HIV-infected infants, it is important to identify where delays in diagnosis occur. This requires that the duration of each step be documented, from blood draw to arrival and testing at the central laboratory to return of results to the clinic and then to the caregiver. Information on each step, however, is not routinely collected and available at all clinics [5]. This study was undertaken to measure the turnaround time for early infant diagnosis in a clinic in rural southern Zambia.

## Materials and Methods

### Ethics Statement

The study was approved by the Ministry of Health of the Government of Zambia, the Institutional Review Board of Macha Research Trust and the Institutional Review Board of the Johns Hopkins Bloomberg School of Public Health. As the study was conducted retrospectively, informed consent was not obtained from participants; however, all data were anonymized prior to analysis.

### Study Population and Setting

This study was conducted at the HIV clinic at Macha Hospital in rural Southern Province, Zambia. Macha Hospital is located in Choma District approximately 80 kilometers from the nearest town of Choma and 350 kilometers from Lusaka by road. The catchment area of Macha Hospital is populated by traditional villagers living in small, scattered homesteads. Macha Hospital is administered by Brethren in Christ Church but functions within the healthcare system of the Ministry of Health. The hospital serves as a district-level referral hospital for rural health centers within an 80 kilometer radius, serving a population of over 150,000 persons. Since 2005, Macha Hospital has provided programs for the prevention of maternal-to-child HIV transmission (PMTCT) and for the care and treatment of HIV-infected individuals [11]–[13]. HIV-infected pregnant women [14] and children [3] receive care and treatment according to Ministry of Health and WHO guidelines.

### Early Infant Diagnosis in Zambia and at Macha Hospital

In 2008, the Zambian Ministry of Health issued new guidelines recommending initiation of ART for all infants younger than 12 months of age and established three national laboratories qualified to perform early infant diagnosis [15]. Two laboratories were established in the capital city of Lusaka at the University Teaching Hospital and Kalingalinga Clinic, and one in Ndola at Arthur Davison Hospital. HIV-exposed infants were recommended to be tested by HIV PCR at 6 weeks and, since 2010, at 6 months of age. If negative at both time points, HIV antibody testing was recommended at 12 and 18 months of age or 6 weeks after cessation of breastfeeding [16].

The early infant diagnosis program at Macha Hospital began in 2008 and is administered through the HIV clinic. HIV-infected women and their exposed infants receive HIV counseling from clinic staff and are advised to have infants tested by HIV PCR at 6 weeks and 6 months of age. Blood is collected from infants by heel stick and stored as dried blood spots (DBS) in the laboratory at the HIV clinic. Batches of specimens are transported to Lusaka in a hospital vehicle for testing by polymerase chain reaction (PCR) at the University Teaching Hospital. Efforts are made to transport specimens once every month. Test results are transported back to the clinic by the hospital vehicle, logged as returned and stored in a binder in the laboratory at the HIV clinic. As this process can be lengthy, caregivers are advised to return to the clinic three months after specimen collection for the test results. For infants who are HIV PCR positive, attempts are made by clinic staff to trace the caregivers in their homes and request that they bring the child back to the clinic as soon as possible. When caregivers return for their appointment, clinic staff review the binder to check if the results were returned. If the results are not found, caregivers are requested to confirm with the laboratory that results are not available and are advised to return in another three months. A shorter interval may be scheduled if the child is due for their 6-month test, the child did not receive an appropriate PMTCT regimen, or if the child has clinical evidence of HIV infection. If the results are available and the infant is HIV PCR negative or the test was invalid, the testing procedures are repeated at 6 months of age. If the HIV PCR test is negative or invalid at 6 months of age, the infant is recommended to return at 12 and 18 months of age or 6 weeks after cessation of breastfeeding (breastfeeding is universal in this community and children are weaned at a median age of 18 months [unpublished data]) for an HIV antibody test. If the results are available and the infant is HIV PCR positive, the caregiver is counseled to have their child begin ART and another DBS specimen is collected for confirmatory testing.

### Study Procedures and Analysis

To assess the turnaround time for early infant testing, a chart review was conducted at the Macha HIV clinic. All DBS specimens collected between August 2010 and March 2012 were abstracted from the laboratory log book. Information routinely collected from the laboratory log book included clinic number, sex, date of birth, date of specimen collection, date of specimen arrival at the laboratory in Lusaka, and date of testing. For study purposes, clinic staff were requested to record the date test results were returned to the HIV clinic in the log book.

For infants who had DBS specimens collected, medical charts were reviewed to confirm the infant’s sex and date of birth, to collect information on maternal and infant receipt of drugs for PMTCT, and to document the date results were returned to the caregiver. When test results were not recorded in the chart to indicate they were provided to the caregiver, the date of the next clinic appointment after results were returned to the HIV clinic was recorded (used for 13% of dates). For infants whose test results were returned to the caregiver more than 4 months after collection, reasons for potential delays were evaluated. Four months was selected as the threshold due to the three-month interval between scheduled appointments. For infants with a positive test result, the date of ART initiation was recorded. Medical charts were reviewed until November 2012 when data collection was stopped.

Data were entered in duplicate using EpiInfo (Centers for Disease Control and Prevention) and analyses were conducted in SAS for Windows version 9.3 (SAS Institute Inc., Cary, NC). Median turnaround times were calculated for the overall process (from blood draw to return of results to the caregiver) and for each step of the process. Median turnaround times for HIV PCR positive and negative test results were compared using the Wilcoxon rank sum test.

## Results

**Figure 1:**
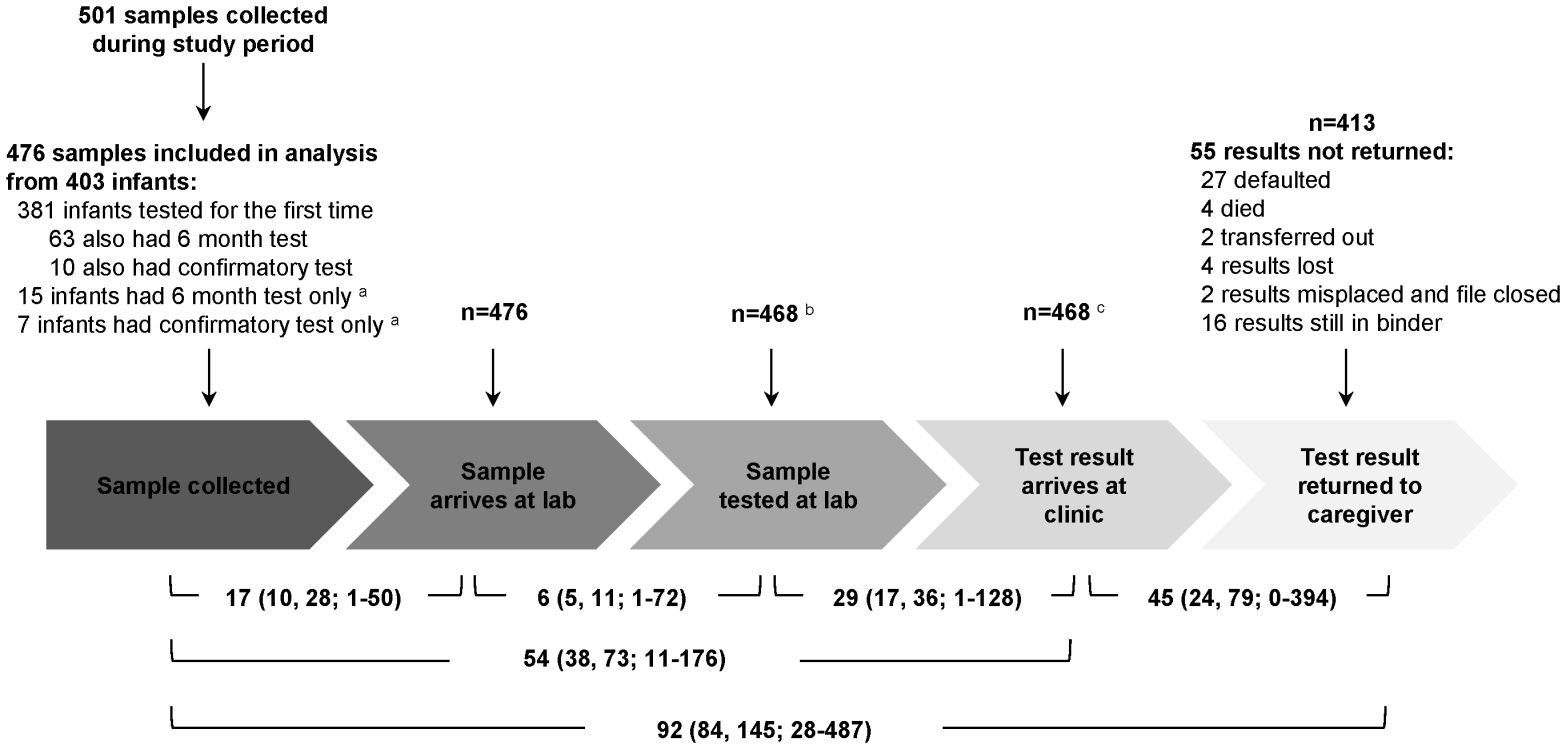
Median turnaround time (IQR; range) in days for early infant diagnosis in rural Zambia, 2010–2012. ^a^These infants were first tested before the study began; ^b^Eight samples were lost at the laboratory; ^c^Six results were missing date of return.

Between August 1, 2010 and March 1, 2012, 501 DBS specimens were collected and sent for testing. Medical records were found for 476 (95%) corresponding specimens and were included in the analysis (Figure 1). The 476 specimens were collected from 403 infants. During the study period, 381 infants had a scheduled “6-week” test or were tested at the clinic for the first time at an older age. The median age of infants at the “6-week” or first test during the study period (n = 381) was 8.1 weeks (interquartile range [IQR]: 6.4, 18.4) (Table 1). Sixty-three of these children also had a “6-month” test and ten had a confirmatory test during the study period. Seven additional infants had a confirmatory test during the study period following a positive test prior to the study period, and 15 infants had a “6-month” test following a “6-week” negative test prior to the study period. The median age of infants at the “6-month” test (n = 78) was 7.0 months (IQR: 6.4, 7.8) and 8.8 months (IQR: 7.0, 12.7) at the confirmatory test (n = 17). The majority of mothers (80%) and children (67%) received PMTCT. Eighteen percent of children received no PMTCT and were born to mothers who had also not received any PMTCT during pregnancy or at birth.

**Table 1:** Characteristics of HIV-exposed infants receiving early infant diagnostic testing in rural southern Zambia, 2010–2012.

	Total N	N (%)
Total HIV-exposed infants	403	
Median age (IQR) in weeks at 6 week (or first ever) test during study period	381	8.1 (6.4, 18.4)
Median age (IQR) in months at 6 month test during study period	78	7.0 (6.4, 7.8)
Median age (IQR) in months at confirmatory test during study period	17	8.8 (7.0, 12.7)
Male	402	212 (53%)
Mother received PMTCT	402	321 (80%)
Highly active ART^a^		205 (64%)
Short course ART		106 (33%)
Single dose NVP		5 (2%)
Missing information		5 (2%)
Infant received PMTCT	390	269 (67%)
Single dose NVP only		14 (5%)
Single dose NVP plus AZT (7 or 28 days)^b^		80 (29%)
AZT (7 or 28 days)^b^ only		22 (8%)
NVP (6 weeks or through breastfeeding)^b^		137 (51%)
AZT (7 or 28 days) plus NVP (6 weeks or through breastfeeding)^b^		8 (3%)
Missing information		8 (3%)

ART: antiretroviral therapy; AZT: zidovudine; IQR: interquartile range; NVP: nevirapine; PMTCT: prevention of mother-to-child transmission;

a The timing of the start of highly active ART during pregnancy was variable.

b Reported duration of AZT and NVP was variable.

Test results were available for 465 (98%) of the 476 specimens: 58 (12%) were HIV PCR positive, 386 (83%) were HIV PCR negative and 21 (5%) were invalid (Table 2). Test results were available for 373 of the 381 infants who underwent their first HIV testing during the study period, with a similar distribution across positive and negative results (Table 2). Seventy-three infants were tested a second time during the study period. Of the 42 infants with an initial positive result, ten had confirmatory testing during the study period and eight (80%) were confirmed HIV PCR positive (two were HIV PCR negative). Of the 313 infants with an initial negative result, 54 had “6-month” or second tests during the study period and 49 (91%) remained HIV PCR negative (one was HIV PCR positive).

**Table 2:** Results of early infant testing during the study period in rural Zambia, 2010–2012.

N for first test		381 infants tested for the first time during study period at 6 weeks of age or older				15 infants with 6 month test only during study period		7 infants with confirmatory test only during study period
Results from first test		Positive	Negative	Invalid	Missing	Negative	Missing	Positive
		42 (11%)	313 (82%)	18 (5%)	8 (2%)^b^	14 (93%)	1 (7%)^a^	7 (100%)
**N for second test**		10 confirmatory tests	54 tests at 6 months	7 tests at 6 months	2 tests at 6 months			
**Results from second test:** **6 month or confirmatory**	**Positive**	8 (80%)	1 (2%)	0	0			
	**Negative**	2 (20%)	49 (91%)	6 (86%)	2 (100%)			
	**Invalid**	0	3 (5%)	0	0			
	**Missing**	0	1 (2%)^c^	1 (14%)^a^	0			

a Result lost at the central laboratory.

b Six results lost at the central laboratory; two results returned but not documented in file.

c Result returned but not documented in file.

Among the 403 infants tested during the study period, test results differed significantly by maternal receipt of PMTCT. Among infants born to mothers who received highly active ART of any duration during pregnancy (n = 205), four (2%) were PCR positive compared to 14 (13%) among infants born to mothers who received short course ART (n = 106) or single-dose nevirapine (n = 5) and 30 (37%) among infants born to mothers who did not receive PMTCT (n = 81) (p = <0.0001). Among the 42 infants who were PCR positive at the “6-week” visit during the study period, 28 (67%) initiated ART by the end of data collection (38% initiated ART on the day results were given; the remainder initiated ART within 10–96 days). Among the 14 infants who did not initiate ART, only two were still in care (3 died, 2 transferred to another clinic, and 7 defaulted) and were diagnosed shortly before the end of data collection.

Turnaround time for each step of the process was measured for each of the 476 specimens (Figure 1). The median time from blood draw to arrival at the central laboratory was 17 days (IQR: 10, 28). For the 468 specimens tested, the median time from arrival at the central laboratory to testing was 6 days (IQR: 5, 11). The median time from testing at the central laboratory to return of the results to the Macha HIV clinic (n = 462; six results were missing a date of return to the clinic) was 29 days (IQR: 17, 36).

Four hundred and thirteen results were returned to the caregiver (87%), with a median time from arrival of the results to the Macha HIV clinic to return of the results to the caregiver (n = 407; six results were missing a date of return to the clinic) of 45 days (IQR: 24, 79). Among the 55 results not returned, reasons included the caregiver defaulting (n = 27; 49%), infant death (n = 4; 7% [2 PCR positive and 2 PCR negative results]), transfer to another clinic (n = 2; 4%), results lost at the clinic (n = 4; 7%), results misplaced and not found prior to discharge from the clinic (n = 2; 4%), and results not yet reported to the caregiver by the end of data collection (n = 16; 29%; median time between arrival of results at clinic and end of data collection: 10.5 months [IQR: 8.8, 13.4]).

For the entire process, the median time from blood draw to arrival of the results at the clinic was 54 days (IQR: 38, 73; n = 462) and to return the results to the caregiver was 92 days (IQR: 84, 145; n = 413). Turnaround times from blood draw to return of results to the caregiver were shorter for specimens that were PCR positive compared to PCR negative or invalid specimens (PCR positive: 85 days [IQR: 70, 113; range: 28–343]; PCR negative or invalid: 92 days [IQR: 84, 146; range: 41–487]), although this result was not statistically significant (p = 0.08). This shorter turnaround time was primarily due to a shorter time between return of results to the clinic and return of results to the caregiver (PCR positive: 30 days [IQR: 15, 55; range: 1–271]; PCR negative: 46 days [IQR: 27, 83; range: 0–394]; p = 0.003).

Among the 413 specimens for which results were returned to the caregiver, 167 (40%) had turnaround times greater than 4 months and 121 (72%) were evaluated for potential delays. Reasons for delay included results not yet returned at the time of the scheduled clinic visit or scheduling of clinic appointments at intervals other than the standard three months (n = 40; 33%), missed appointments by caregivers (n = 19; 16%), clinic error (results not found by clinic staff despite being logged as returned to the HIV clinic; n = 60; 50%), and a combination of missed appointments by the caregiver and clinic error (n = 2; 2%).

## Discussion

In this study of turnaround times for early infant HIV diagnosis test results in a rural clinic in southern Zambia, the median time from sample collection to return of the results to the caregiver was 92 days. Significant delays occurred in getting results back to the clinic from the laboratory and in getting the results to the caregiver. These findings suggest that reductions in turnaround time can be achieved by developing strategies to improve communication between the laboratory and the clinic, and between the clinic and caregiver.

Similar to other rural settings, the clinic at Macha Hospital transported samples to a central laboratory in Lusaka approximately once a month and retrieved results from the laboratory when the results and transportation were available. As the duration of this process was variable, healthcare providers scheduled clinic visits at three-month intervals to avoid having caregivers travel to the clinic before test results were available. The three-month interval also coincided with clinic visits for caregivers to minimize the travel burden [13]. The turnaround time from sample collection to return of results to the clinic of 54 days indicates that the three-month interval is justified and is similar to findings from other studies in Zambia (44.2 days) [8] and Uganda (35–57 days) [10].

The longest interval for the testing process was from testing at the central laboratory to return of the results to the clinic. This interval was approximately one month, suggesting significant delays and a potential step that could be shortened. In a pilot study in Zambia, use of an SMS-based system that enabled central laboratories to report results to clinics in advance of receiving the hard copy succeeded in reducing the turnaround time from 44.2 to 26.7 days [8]. Using a similar SMS-based system in Uganda, 8 of 10 pilot sites reduced the turnaround time to below the target of 30 days, with SMS results reaching the clinics 17 to 27 days prior to the hard copy [10]. Similar results were found in Kenya and Nigeria [9]. Following the pilot study, the Government of Zambia began implementing the SMS-based message system in rural districts with national scale-up planned by 2014 [8], [17]. In January 2013, an SMS-based system was established at the Macha HIV clinic and is expected to decrease the time to return test results from the central laboratory in Lusaka.

Once the results were received at the clinic, a median of 45 days passed to return the results to the caregiver, for a total turnaround time of 92 days. Few other studies have documented the duration of the entire process for comparison. A study in neighboring rural districts in Zambia measured a total turnaround time of 66.8 days [8], and a study in rural Tanzania found a total turnaround time of 5 weeks for HIV-infected infants and 10 weeks for HIV-uninfected infants [7]. In the current study, efforts were made to trace caregivers of infants with positive test results to return to the clinic earlier than scheduled, resulting in a shorter time to return results to the caregiver (30 days) and a shorter total turnaround time (85 days) for HIV PCR positive children.

Forty percent of results returned to the caregiver took longer than four months. Some delays were due to errors on the part of the caregiver in missing clinic appointments but the majority of delays were due to errors on the part of the clinic in misplacing or failing to locate results when caregivers returned for their appointments. The operational tasks of early infant diagnosis have increased the burden on an already busy and understaffed clinic [18]. Without staff dedicated to early infant diagnosis, delays will likely continue to occur.

Attrition in PMTCT and HIV care programs presents an additional challenge to diagnosing HIV-infected infants and enrolling them in care [19], [20]. Even after testing HIV-exposed infants, a significant proportion of caregivers do not return for the results. At PMTCT clinics in Tanzania and Malawi, only 55% and 60% of caregivers returned for test results, respectively [7], [21]. Importantly, more caregivers of infants testing positive returned, thereby increasing the likelihood that HIV-infected infants would enroll in care and receive treatment. In Malawi, 58% of infants who tested positive started ART [21] and in a report of Namibia’s national program this proportion reached 70% in 2008 [5]. In the current study conducted at a single HIV clinic, attrition was low: 86% of caregivers returned for results and 67% of infants who tested positive started ART by the end of the study.

In addition to decreasing the time to return results to the clinic, an SMS-based system has the potential to decrease the time to return results to the caregiver and increase retention [8]. Caregivers with access to mobile phones could be notified that results are available at the clinic and community health workers could be notified to contact caregivers without access to mobile phones. Direct communication with caregivers about the availability of results could get them to return to the clinic earlier than scheduled, ensure that results are provided at clinic visits, and potentially decrease the likelihood of defaulting.

## Conclusions

Delays in early infant diagnosis were identified in returning results from the central laboratory to the clinic and from the clinic to the caregiver. In this rural setting in sub-Saharan Africa, transportation presents a challenge for both clinics and caregivers. For the clinic, the need to transport samples to and results from the central laboratory creates delays, particularly in determining the availability of results at the clinic to communicate to the caregiver. For the caregiver, long travel times to the clinic necessitate scheduling appointments at three-month intervals to coincide with their own clinic visits. More efficient communication is needed so caregivers can be provided test results sooner, potentially resulting in earlier treatment initiation and better outcomes for HIV-infected infants.
